# Reduction of dislocations in α-Ga_2_O_3_ epilayers grown by halide vapor-phase epitaxy on a conical frustum-patterned sapphire substrate

**DOI:** 10.1107/S2052252521003389

**Published:** 2021-04-28

**Authors:** Hoki Son, Ye-ji Choi, Soon-Ku Hong, Ji-Hyeon Park, Dae-Woo Jeon

**Affiliations:** a Korea Institute of Ceramic Engineering and Technology, 15-5, Jinju-si, Gyeongsangnam-do 52851, Republic of Korea; b Department of Material Science and Engineering, Korea university, Seoul 02841, Republic of Korea; c Department of Materials Science and Engineering, Chungnam National University, Daejeon, 34134, Republic of Korea

**Keywords:** α-Ga_2_O_3_, ultra-wide bandgaps, halide vapor-phase epitaxy, epitaxial lateral overgrowth, crystallization, crystal growth, crystal design

## Abstract

The compound α-Ga_2_O_3_ is an ultra-wide-bandgap semiconductor and possesses outstanding properties such as a high breakdown voltage and symmetry compared with other phases. It has been studied for applications in high-performance power devices. However, it is difficult to obtain a high-quality thin films because α-Ga_2_O_3_ can only grow heteroepitaxially, which results in residual stress generation owing to lattice mismatch and thermal expansion between the substrate and α-Ga_2_O_3_. To overcome this, α-Ga_2_O_3_ was grown on a conical frustum-patterned sapphire substrate by halide vapor-phase epitaxy. The surface morphology was crack-free and flat. The α-Ga_2_O_3_ grown on a frustum-patterned substrate and a conventional sapphire substrate at 500°C exhibited full-width at half-maxima of 961 and 1539 arcsec, respectively, for 10–12 diffraction. For the former substrate, lateral growth on the pattern and threading dislocation bending towards the pattern suppressed the propagation of threading dislocations generated at the interface, which reduced the threading dislocation propagation to the surface by half compared with that on the latter conventional substrate. The results suggest that conical frustum-patterned sapphire substrates have the potential to produce high-quality α-Ga_2_O_3_ epilayers.

## Abstract 2

Low dislocation density of α-Ga_2_O_3_ grown on conical frustum-patterned sapphire substrate (CF-PSS) has been studied. The threading dislocation propagation path of α-Ga_2_O_3_ on CF-PSS was observed.

## 1. Introduction   

To improve the performance of power devices, materials with outstanding physical properties as well as improved device fabrication processes are essential. For decades silicon, which drives power devices, has contributed to improving their performance through various processes; however, the theoretical limit of silicon’s role in power devices is clearly distinguished by material properties (Higashiwaki, Murakami*, et al.*, 2016 ▸). To solve this problem, studies on various materials are underway, and recently, ultra-wide-bandgap materials have attracted considerable attention (Higashiwaki, Sasaki *et al.*, 2016 ▸; Pearton *et al.*, 2018 ▸). Ultra-wide-bandgap materials include aluminium nitride, boron nitride, diamond and gallium oxide (Ga_2_O_3_) and offer a bandgap greater than 3.4 eV.

According to the power semiconductor roadmap, Ga_2_O_3_ is considered to be the next-generation semiconductor material (Higashiwaki, Sasaki *et al.*, 2016 ▸; Oda *et al.*, 2016 ▸). Ga_2_O_3_ has five phases (α, β, γ, ɛ and δ) that can be selected by growth conditions such as temperature, working pressure and growth method (Xue *et al.*, 2018 ▸). It has a wide bandgap of 4.5–5.2 eV and a high breakdown voltage (8 MV cm^−1^ and 10 MV cm^−1^). It also exhibits properties such as high stability at high temperature and voltage, high dielectric constant (∼10) and low electron mobility (Leach *et al.*, 2019 ▸). Also, Baliga’s figure of merit (FOM), which represents the performance of a power device, is very high. This material has the potential to be used in various devices, for example, in field-effect transistors (FETs), Schottky barrier diodes (SBDs) and UV optical devices (Oda *et al.*, 2016 ▸; Sasaki *et al.*, 2013 ▸; Ghose *et al.*, 2017 ▸). Research on β-Ga_2_O_3_ is more widespread compared with the other phases. β-Ga_2_O_3_ has a monoclinic structure and is the most stable phase, and liquid-phase growth is possible (Mu *et al.*, 2017 ▸; Aida *et al.*, 2008 ▸) which can yield high-quality substrates that are also inexpensive.

In addition, homoepitaxial growth is possible, and high-performance equipment can be manufactured (Murakami *et al.*, 2014 ▸). It has been reported that metal semiconductor FETs and SBDs containing β-Ga_2_O_3_ have a breakdown voltage of 531 V and an on-resistance of 0.1 mΩ cm^2^ (Oda *et al.*, 2016 ▸; Xue *et al.*, 2018 ▸). α-Ga_2_O_3_, which has improved characteristics, displays the widest bandgap, breakdown voltage, electron mobility and Baliga’s FOM. These characteristics are dominant in α-Ga_2_O_3_ compared with β-Ga_2_O_3_ (Neal *et al.*, 2017 ▸). In addition, α-Ga_2_O_3_ has a corundum structure, which forms a ternary system with indium oxide and aluminium oxide, enabling both bandgap engineering to produce a desired wavelength and function engineering to improve the characteristics using transition metals (Cr, Fe, V, Ti) (Feneberg *et al.*, 2018 ▸). However, α-Ga_2_O_3_ undergoes a phase transition at high temperature (>700°C) involving a metastable state, which is an undesirable disadvantage; hence, the substrate cannot be fabricated by liquid-phase growth, and only heteroepitaxy growth occurs (Oshima *et al.*, 2015 ▸; Son *et al.*, 2019 ▸).

Heteroepitaxy generates residual stress because of the difference in the thermal expansion coefficient and the lattice constant between the starting substrate and the epilayer grown (Cariou *et al.*, 2016 ▸). Dislocations are created inside the film grown in order to relax the residual stress generated. This degrades the performance of the devices and various methods for improving the quality of thin films have been explored, one of which is the use of a buffer layer and epitaxial lateral overgrowth (ELOG) (Jinno *et al.*, 2018 ▸, 2016 ▸; Oshima *et al.*, 2019 ▸). The buffer layer is grown between the starting substrate and the growth film to decrease the difference in the lattice constants, thereby reducing the residual stress. An α-(AlGa)_2_O_3_ layer using an aluminium alloy was used as the buffer layer in α-Ga_2_O_3_. As a result, the threading dislocation density (TDD) of α-Ga_2_O_3_ decreased by more than one order of magnitude compared with that without a buffer layer (Jinno *et al.*, 2016 ▸). However, growth of the ternary buffer layer is difficult, and the layer material may diffuse into the epilayer and increase the impurity concentration (Chaaben *et al.*, 2016 ▸).

In ELOG, growth occurs only in periodically fabricated seed regions followed by coalescence. This method decreases the TDD observed at the surface because the interface between the epilayer and the substrate is reduced to suppress the occurrence of dislocations, and the dislocations propagating to the surface are bent laterally (Oshima *et al.*, 2019 ▸).

In this study, α-Ga_2_O_3_ epilayers were grown on a conical frustum-patterned sapphire substrate (CF-PSS) by halide vapor-phase epitaxy (HVPE). The α-Ga_2_O_3_ epilayers grown on CF-PSS were examined and compared with those grown on the conventional sapphire substrate. The use of the CF-PSS decreases the threading dislocations (TDs) by promoting lateral growth on patterns and bending in the pattern, as observed by transmission electron microscopy (TEM).

## 2. Methods   

The α-Ga_2_O_3_ epilayers were grown by HVPE on a conventional sapphire substrate (CSS) and a CF-PSS. HVPE was employed with an atmospheric horizontal hot wall acting as a resistor heater and divided into a source zone and a growth zone. Liquid gallium metal, as a group III precursor, was placed in the source zone. The liquid Ga metal reacts with the hydro­chloric acid gas to produce gallium monochloride (GaCl) and gallium trichloride (GaCl_3_). The temperature of the source zone was fixed at 470°C, and GaCl was generated as the major reactant (Cariou *et al.*, 2016 ▸). GaCl reacts with oxygen as a group VI precursor in the growth zone and is synthesized as α-Ga_2_O_3_ on substrates such as CSS and CF-PSS. The temperature of the growth zone was maintained at 500°C. Nitro­gen was used as the main carrier gas. The total gas flow was fixed at 5 l min^−1^.

The thickness of the α-Ga_2_O_3_ epilayers was approximately 3 µm, and the growth rate was 6 µm h^−1^. The pattern size in the CF-PSS was 1.1 µm in top circle width and 0.6 µm in height. The surface and cross-sectional morphologies of the grown α-Ga_2_O_3_ epilayers were observed by field-emission scanning electron microscopy (FE-SEM). The surface roughness was measured by atomic force microscopy (AFM). The structure and crystal quality of the epilayers were investigated by θ–2θ scan and ω rocking curve measurements for the 0006 and 10–12 diffractions using high-resolution X-ray diffraction with Cu *K*α_1_ radiation of 1.54 Å wavelength. The X-ray diffractometer consisted of a line source, a graded parabolic (multilayer) mirror, a four-bounce symmetric Ge (440) monochromator and a two-bounce channel-cut Ge (220) analyzer in front of the detector. Cross-sectional TEM was performed to observe the TDs in the α-Ga_2_O_3_ epilayer.

## 3. Results and discussion   

**Figure 1:**
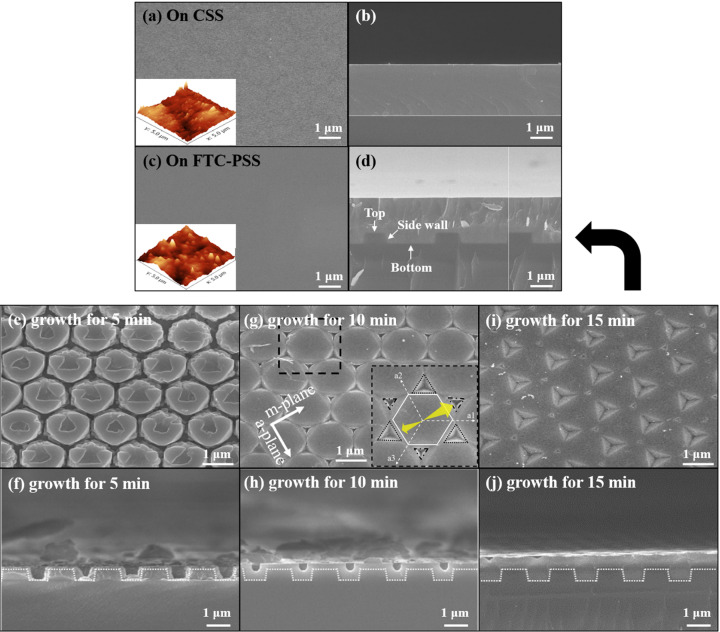
FE-SEM and AFM (inset) images of surfaces and cross-sections of the α-Ga_2_O_3_ epilayers grown on (*a*)–(*b*) CSS and (*c*)–(*d*) CF-PSS. Time evolution of α-Ga_2_O_3_ epilayers on CF-PSS at growth times of (*e*)–(*f*) 5, (*g*)–(*h*) 10 and (*i*)–(*j*) 15 min.

The surface and cross-sectional FE-SEM images of the α-Ga_2_O_3_ epilayers grown on CSS and CF-PSS are shown in Fig. 1 ▸. The surface morphologies of α-Ga_2_O_3_ epilayers grown on CSS and CF-PSS were flat and crack-free. The root mean square roughness values of α-Ga_2_O_3_ epilayers grown on CSS and CF-PSS measured by AFM were 7.3 and 5.9 nm, respectively, and the surface of the α-Ga_2_O_3_ epilayer on CF-PSS was more uniform.

The morphology of the α-Ga_2_O_3_ epilayer grown on CF-PSS was observed with increasing growth time, as shown in Figs. 1 ▸(*e*)–1(*j*). During the initial growth time of 5 min [Figs. 1 ▸(*e*) and 1(*f*)], all areas of the patterns were covered with Ga_2_O_3_ grains. The difference in growth rate according to the growth direction was not noticeable. At a growth time of 10 min [Figs. 1 ▸(*g*) and 1(*h*)], the space between the patterns was filled due to *c*-axis growth at the bottom and lateral growth at the sidewall without air voids. In particular, we confirmed that the lateral growth on the top region of the pattern occurred preferentially in the *m*-plane direction, and among them, the lateral growth rates were relatively high at three *m*-planes with a 120° angle (shown in the inset). In the top region of the patterns, small inversed-triangular-pyramidal shapes were regularly observed at the surface at a growth time of 15 min [Figs. 1 ▸(*i*) and 1(*j*)]. This is the result of lateral growth in six *m*-plane directions because of the difference in the high growth rates among the specific three *m*-plane directions. Because of this difference in growth rate, by employing the CF-PSS, the areas grown in the *m*-plane directions were merged and additional growth time was required for a smooth surface.

**Figure 2:**
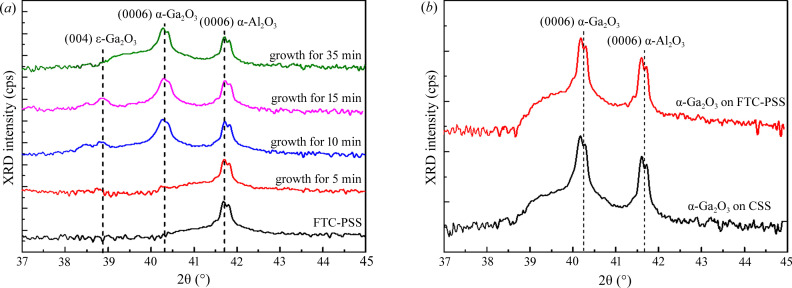
XRD spectra from θ–2θ scans of the α-Ga_2_O_3_ epilayers grown on CSS and CF-PSS; (*a*) α-Ga_2_O_3_ epilayers on CF-PSS at different growth times for 5–35 min and (*b*) after growth.

However, the results suggest there is potential for growth of the α-Ga_2_O_3_ epilayer with improved surface morphology and that lateral growth was promoted in the *m*-plane direction compared with the CSS. XRD was used to investigate the crystal structure of the epilayer. Fig. 2 ▸(*a*) shows the XRD θ–2θ scan spectra of the α-Ga_2_O_3_ epilayers grown on CF-PSS for 5, 10, 15 and 35 min. The 0006 diffraction peak of the α-Ga_2_O_3_ epilayer was very small at a growth time of 5 min, which represents the initial stage of growth. Additionally, the sapphire peak was the major peak, similar to the CF-PSS. At a growth time of 10 min, the 0006 diffraction peak of the α-Ga_2_O_3_ epilayer and the 004-diffraction peak of ɛ-Ga_2_O_3_ were observed. We assumed that the α-phase was grown at the top and bottom of the CF-PSS, and the ɛ-phase was grown on the sidewall of the CF-PSS. In a previous report, Shapenkov *et al.* (2020 ▸) confirmed that an α-Ga_2_O_3_ epilayer was grown on the top of the pattern, and an ɛ-Ga_2_O_3_ epilayer was grown on the sidewall of the pattern. The 004 diffraction peak position of the α-Ga_2_O_3_ epilayer was observed at 38.85° (JCPDS No. 06–0509). The intensity of the 0006 diffraction peak of the α-Ga_2_O_3_ epilayer increased with continuous growth. However, the 004 diffraction peak of the ɛ-Ga_2_O_3_ epilayer disappeared. As the lateral growth of the α-phase progressed, it was thought that the ɛ-phase, which would have grown initially in the pattern side, was blocked.

Fig. 2 ▸(*b*) shows the XRD θ–2θ scan spectra of the α-Ga_2_O_3_ epilayers grown on CSS and CF-PSS. The stress-free 0006 diffraction peak position of α-Ga_2_O_3_ was 40.24° (JCPDS No. 06–0503). The lattice constants of the α-Ga_2_O_3_ epilayer were *a* = 4.9825 and *c* = 13.433 Å, and those of the α-Al_2_O_3_ substrate were *a* = 4.765 and *c* = 13.001 Å. The lattice mismatch between the α-Ga_2_O_3_ epilayer and α-Al_2_O_3_ substrate was 4.6% on the *a* axis and 3.3% on the *c* axis, which is relatively large. The 0006 diffraction peak of α-Ga_2_O_3_ epilayers grown on CSS and CF-PSS was observed at 40.18°. This peak position was shifted to a lower angle compared with that of the strain-free α-Ga_2_O_3_ epilayers. The lattice constants of both α-Ga_2_O_3_ epilayers were calculated as *a* = 4.9799 and *c* = 13.455 Å. This result indicates that both α-Ga_2_O_3_ epilayers were in a slightly compressive stress state. This compressive stress was caused by the difference in the coefficient of thermal expansion. The thermal expansion coefficients of the sapphire substrate and α-Ga_2_O_3_ epilayer were 8.6 × 10^−6^ and 1.1 × 10^−5^ K^−1^, respectively (Higashiwaki & Fujita, 2020 ▸). As the α-Ga_2_O_3_ epilayer grew and was cooled, compressive stress was generated, resulting in a peak shift to a low angle.

**Figure 3:**
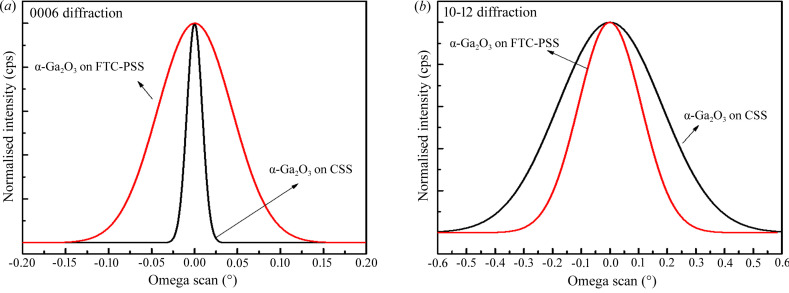
X-ray omega rocking curves of the α-Ga_2_O_3_ epilayers grown on CSS and CF-PSS; (*a*) 0006 diffraction and (*b*) 10–12 diffraction.

Fig. 3 ▸ shows the typical X-ray rocking curves (XRCs) obtained for the α-Ga_2_O_3_ epilayers on CSS and CF-PSS. The full width at half-maximum (FWHM) of the 0006 diffraction peak is symmetric with respect to the screw dislocation, and the FWHM of the 10–12 diffraction peak is asymmetric with respect to the edge and mixed dislocations. The FWHMs of the 0006 and 10–12 diffraction peaks of the α-Ga_2_O_3_ epilayers on CSS were 75 and 1539 arcsec, respectively. In our previous study, the FWHMs of the 0006 and 10–12 diffraction peaks of 1 µm α-Ga_2_O_3_ epilayers on CSS were 27 and 3254 arcsec, respectively (Son & Jeon, 2019 ▸). The FWHM of the 0006 diffraction peak increased slightly, whereas the FWHM of the 10–12 diffraction peak decreased significantly. It appears that the thickness of the α-Ga_2_O_3_ epilayer increased, and the TDs generated at the interface merged while being directed to the surface. On the other hand, the FWHMs for the 0006 and 10–12 diffractions of the α-Ga_2_O_3_ epilayers on CF-PSS were 368 and 961 arcsec, respectively. Compared with the α-Ga_2_O_3_ epilayers on CSS, the FWHMs of the 0006 and 10–12 diffraction peaks increased and decreased, respectively. Chen *et al.* (2018 ▸) reported that periodic patterns of the sapphire substrate were beneficial for suppressing grain twisting when the adjacent grains coalesce. However, CSS did not favour grain twisting, though it was advantageous to suppress the tilt of the grain. It is assumed that the FWHMs of the 0006 and 10–12 diffraction peaks were affected by the growth on the pattern.

**Figure 4:**
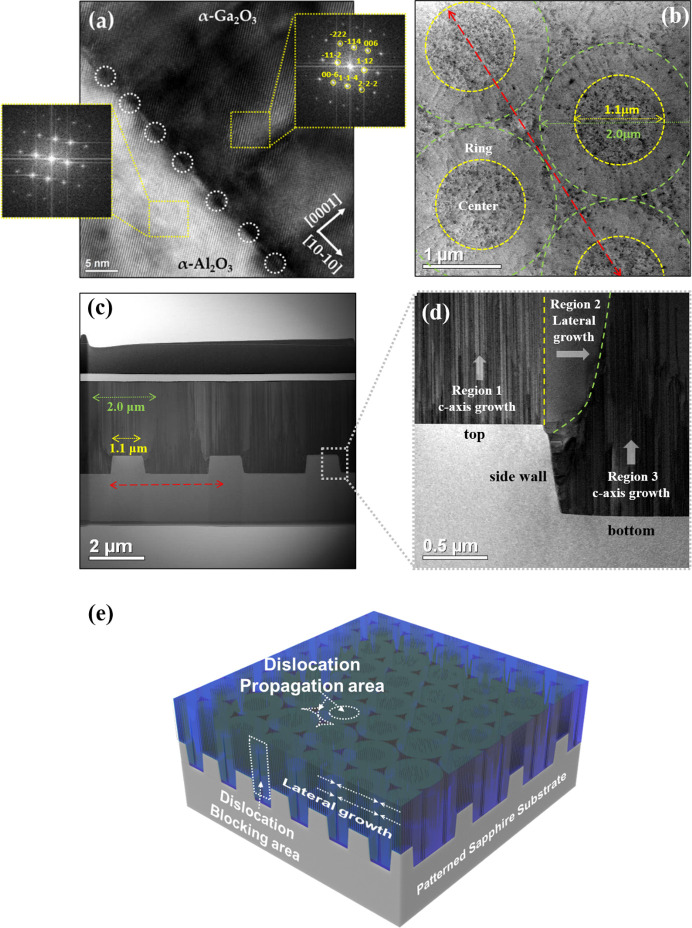
(*a*) High-magnification cross-sectional TEM image of the α-Ga_2_O_3_ epilayers/α-Al_2_O_3_ interface viewed along [11–20] axis. Inset diffraction patterns of α-Ga_2_O_3_ epilayers grown on CF-PSS. (*b*) Plan-view, (*c*)–(*d*) low-magnification cross-sectional TEM image. (*e*) Schematic of the behavior of TD in the α-Ga_2_O_3_ epilayer grown on CF-PSS.

To confirm the effect of CF-PSS on the α-Ga_2_O_3_ epilayer, TEM was carried out. Fig. 4 ▸(*a*) shows a cross-sectional TEM image of the α-Ga_2_O_3_ epilayer–CF-PSS interface observed along the [11–20] zone axes. The dark areas (dashed circle) were periodically observed at the α-Ga_2_O_3_ epilayer–CF-PSS interface, indicating misfit dislocations (MDs) on the α-Ga_2_O_3_ epilayer–sapphire interface. MDs occur when the length of 20 crystal cells of the α-Ga_2_O_3_ epilayer with a large lattice constant coincides with that of 21 crystal cells of α-Al_2_O_3_ with a small lattice constant (Kaneko *et al.*, 2012 ▸). MDs were generated to alleviate the in-plane compressive strain caused by the difference in lattice parameters between α-Ga_2_O_3_ and α-Al_2_O_3_. The inset images (dashed square) in Fig. 4 ▸(*a*) show the electron diffraction patterns for α-Ga_2_O_3_ and sapphire, respectively, corresponding to the corundum structure. The epitaxial relationships between the α-Ga_2_O_3_ epilayer and CF-PSS were (0006) α-Ga_2_O_3_ epilayer∥(0006) sapphire. Figs. 4 ▸(*b*) and 4(*c*) show the plan-view and cross-sectional TEM images of the α-Ga_2_O_3_ epilayer on CF-PSS. The dark spots on the surface indicate the TDs. We can confirm that the end-on strain contrast from TDs on the surface did not appear uniformly, and the densities of TDs were relatively lower in a certain region of the ring shape. The TDD of the ring region was 9 × 10^8^ cm^−2^ and that at the center region was 1.6 × 10^10^ cm^−2^. As a result, the average TDD in the α-Ga_2_O_3_ epilayer was determined to be 8.4 × 10^9^ cm^−2^.

The high-magnification TEM image [Fig. 4 ▸(*d*)] can be divided into three regions according to the distribution of TDs. In regions 1 and 3, the α-Ga_2_O_3_ epilayer growth occurred along the *c* axis, which can be confirmed by the propagation of the TDs generated at the interface. In contrast, the TDs were negligible in region 2. As α-Ga_2_O_3_ was grown in region 1, the lateral growth of α-Ga_2_O_3_ occurred simultaneously, and the width of the lateral growth gradually increased with longer growth times. As a result, the TDs generated in region 3 were significantly decreased (or prevented) by the lateral growth region, and a region with a low density of dark spots developed that can be attributed to the TDs that appeared on the surface. Fig. 4 ▸(*e*) shows a schematic of the growth mechanism of the α-Ga_2_O_3_ epilayer on CF-PSS. The dotted-line rectangle shows the dislocation-blocking area because of the lateral growth. Consequently, we determined that the crystal quality of the α-Ga_2_O_3_ epilayer on CF-PSS was improved compared with that on the CSS owing to the blocking of dislocations by the lateral growth of α-Ga_2_O_3_.

## 4. Conclusions   

We studied a single-crystal α-Ga_2_O_3_ epilayer on CF-PSS using HVPE. The thickness of the α-Ga_2_O_3_ epilayers was approximately 3 µm at a growth temperature of 500°C. The α-Ga_2_O_3_ epilayers grown exhibited slightly in-plane compressive stress because of the lattice mismatch and difference in thermal expansion coefficients between the substrate and α-Ga_2_O_3_. The 10–12 diffraction FWHMs of the α-Ga_2_O_3_ epilayer grown on CF-PSS and CSS were 961 and 1539 arcsec, respectively. The MDs were produced at the interface between the substrate and the α-Ga_2_O_3_ epilayer, as well as in the α-Ga_2_O_3_ epilayer, creating an end-on strain contrast of TDs on the surface of the α-Ga_2_O_3_ epilayer. The average TDDs in the α-Ga_2_O_3_ epilayer on CF-PSS and CSS were 8.4 × 10^9^ and 1.6 × 10^10^ cm^−2^, respectively, both of which exhibited a decrease in TDs. The reduction of TDs was observed differently according to the growth of the α-Ga_2_O_3_ epilayer in the pattern. In the *c*-axis growth, the TDs are the same as those along the growth direction. On the other hand, TDs were negligible during the lateral growth. This lateral growth obstructed the path of the TDs propagating between the patterns to the surface, thus significantly decreasing the TDs appearing on the surface.
